# Debonding‐On‐Demand Polymeric Wound Patches for Minimal Adhesion and Clinical Communication

**DOI:** 10.1002/advs.202202635

**Published:** 2022-08-21

**Authors:** Qiankun Zeng, Fangbing Wang, Ruixuan Hu, Xuyin Ding, Yifan Lu, Guoyue Shi, Hossam Haick, Min Zhang

**Affiliations:** ^1^ School of Chemistry and Molecular Engineering Shanghai Key Laboratory for Urban Ecological Processes and Eco‐Restoration Shanghai Key Laboratory of Multidimensional Information Processing Engineering Research Centre for Nanophotonics and Advanced Instrument (Ministry of Education) East China Normal University Shanghai 200241 China; ^2^ Department of Chemical Engineering and Russell Berrie Nanotechnology Institute Technion – Israel Institute of Technology Haifa 320003 Israel

**Keywords:** clinical communication, ionic conducting elastomer, polymeric adhesive, wound patches

## Abstract

Herein, a multifunctional bilayer wound patch is developed by integrating a debonding‐on‐demand polymeric tissue adhesive (DDPTA) with an ionic conducting elastomer (ICE). As a skin adhesive layer, the DDPTA is soft and adherent at skin temperature but hard and non‐tacky when cooled, so it provides unique temperature‐triggered quick adhesion and non‐forced detachment from the skin. During use, the dense surface of the DDPTA prevents blood infiltration and reduces unnecessary blood loss with gentle pressing. Moreover, its hydrophobic matrix helps to repel blood and prevents the formation of clots, thus precluding wound tearing during its removal. This unique feature enables the DDPTA to avoid the severe deficiencies of hydrophilic adhesives, providing a reliable solution for a wide range of secondary wound injuries. The DDPTA is versatile in that it can be covered with ICE to configure a DDPTA@ICE patch for initiating non‐verbal communication systems by the fingers, leading toward sign language recognition and a remote clinical alarm system. This multifunctional wound patch with debonding‐on‐demand can promote a new style of tissue sealant for convenient clinical communication.

## Graphical abstract

In this work, a debonding‐on‐demand bilayer wound patch is introduced that can serve as a multifunctional platform for wound sealing and clinical communication with the wounded.

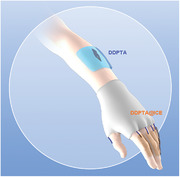

## 1 Introduction

Millions of people annually suffer from mild to life‐threatening tissue injuries resulting from trauma, acute postsurgical wounds, burns, abrasions, and chronic wounds due to circulatory disturbances or diabetes.^[^ ^1^, ^2^ ^]^ Recent years have witnessed huge investments in the exploration of therapeutic materials^[^ ^3^, ^4^ ^]^ and clinical care^[^ ^5^, ^6^ ^]^ for wounds, in response to the serious consequences of tissue injury and the rapid growth of the associated market. Open wounds require immediate closure to stop bleeding, isolate external disturbances, and ultimately provide a proper healing environment.^[^ ^7^, ^8^ ^]^ At the same time, since numerous people around the world have language, visual, hearing, or physical disabilities, rapid and effective non‐verbal clinical communication has become a major challenge in caring for these patients.^[^ ^9^, ^10^ ^]^ Recent advances in adhesives have provided versatile materials for wound treatment and care that can bond firmly with biological tissue to seal wounds or carry communication devices.^[^ ^11^, ^12^ ^]^ For example, Li et al. reported a family of bioinspired adhesives with broad applications ranging from hemostasis and tissue adhesives to wound dressings and tissue repair.^[^ ^13^ ^]^ Wang et al. developed an ultrasoft bioadhesive hydrogel‐integrated brain‐machine interface for electroencephalographic signal acquisition and communication.^[^ ^14^ ^]^ For these applications, the key requirement is that the adhesives should stick firmly to the surrounding tissue and can be removed easily as needed to preclude injury to the skin and the wounds. However, only a few adhesives meet this requirement.

Generally, the choice of adhesive depends on replacement requirements, ease of use and removal, patients’ comfort, harmlessness to surrounding tissue, and cost.^[^ ^14^, ^15^ ^]^ To prevent peeling, most common adhesives bind too strongly, inevitably resulting in local trauma and pain during replacement.^[^ ^16^, ^17^ ^]^ Although a few reversible adhesives have been developed recently, they function under specific or harsh trigger conditions such as extreme pH, UV exposure, or metal ions, which can entail toxic effects.^[^ ^18^, ^19^ ^]^ Successful implementation of adhesives also remains challenging because they bind strongly to the clot on the wound,^[^ ^12^ ^]^ which can lead to wound tearing and secondary bleeding when they are removed.

**Figure 1:**
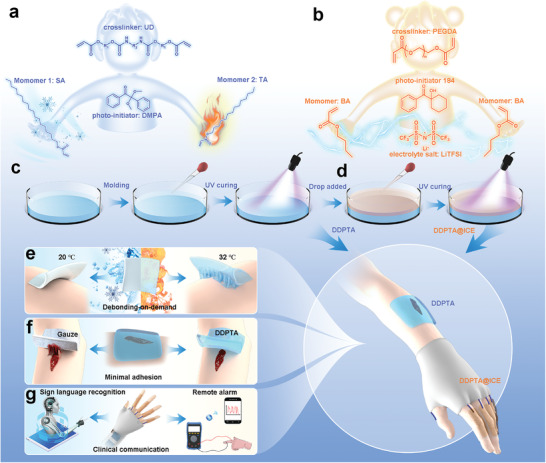
Engineering a multifunctional bilayer polymer wound patch. Molecular building blocks for preparing a) DDPTA and b) ICE. Schematic illustrating the fabrication of c) DDPTA patch and d) DDPTA@ICE patch. Illustrations of our presented wound patch with e) temperature‐response debonding‐on‐demand, uses for f) wound sealing with minimal adhesion, and g) sign language recognition and remote clinical communication.

The ideal adhesive should provide easy debonding on demand and minimal adhesion.^[^ ^20^ ^]^ With this in mind, we present a debonding‐on‐demand polymeric tissue adhesive (DDPTA) for sealing wounds and integrating with conductive material to facilitate the development of non‐verbal communication systems (**Figure** **1**). The DDPTA can be fabricated by incorporating stearyl acrylate (SA, monomer 1) and tetradecyl acrylate (TA, monomer 2), which have different melting temperatures, into a chemically cross‐linked elastomeric network by instant UV‐curing,^[^ ^21^ ^]^ using urethane diacrylate (UD) as cross‐linker and 2,2‐dimethoxy‐2‐phenylacetophenone as photo‐initiator (Figure 1a). The DDPTA is soft and adherent at skin temperature but hard and non‐tacky when cooled, so it allows temperature‐triggered on‐demand adhesion/removal (Figure 1e). It is advantageous that the wound is not reopened during replacement of the DDPTA. This interesting property is attributable to the semicrystalline‐to‐amorphous transition triggered by temperature changes. During use, the dense surface of the DDPTA prevents blood infiltration and reduces unnecessary blood loss with gentle pressing; its hydrophobic matrix helps to repel blood and prevents the formation of clots, thus precluding wound tearing during its removal (Figure 1f). This unique feature enables the DDPTA to avoid the severe deficiencies of hydrophilic adhesives, providing a reliable solution for a wide range of secondary wound injuries.

Taking advantage of the DDPTA's on‐demand adhesion/removal, we further introduced a layer of ionic conducting elastomer (ICE)^[^ ^22^ ^]^ on its surface to form a bilayer DDPTA@ICE patch. We used the same instant UV‐curing process (Figure 1d), mixing butyl acrylate (BA, monomer) with 1‐hydroxycyclohexyl phenyl ketone (photo‐initiator 184), polyethyleneglycol diacrylate (PEGDA, cross‐linker), and lithium bis(trifluoromethanesulfonimide) (LiTFSI, electrolyte salt) (Figure 1b). Non‐verbal communication systems using the fingers were readily constructed on the basis of the resulting DDPTA@ICE patch (Figure 1g). These systems enable sign language to be recognized and provide a remote clinical alarm signal to assist the wounded with gesture communication and remote clinical care. The DDPTA@ICE patch avoids the shortcomings of traditional hydrogel patches (e.g., rapid water loss, low transparency, and difficulty of removal) and offers on‐demand adhesion/removal. Powered by machine learning, a DDPTA@ICE‐based sign language recognition system recognizes different gestures independently by monitoring the resistive response patterns. This can break down communication barriers between nonsigners and signers. To promote its clinical applications, a remote clinical alarm system on the fingers, consisting of a DDPTA@ICE patch, a smartphone, and a Bluetooth‐enabled digital multimeter, provides remote communication between doctors and the wounded. In the event of an emergency or need for help, the wounded simply bend their fingers to increase the resistance signal of the patch sharply and exceed the threshold value, alerting doctors or family members through mobile phone vibrations. Our non‐verbal communication systems are expected to serve as a bridge between the wounded and others and to be invaluable in the transmission of clinical information.

## 2 Results and Discussion

**Figure 2:**
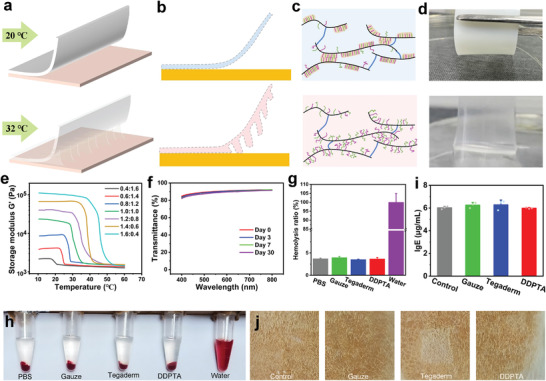
Schematic diagram of a) macroscopic and b) microscopic interfacial behavior of DDPTA during peeling. c) Schematic illustrating the linear alkyl chains of DDPTA in semicrystalline (20 °C) and amorphous (32 °C) phases. d) Photos of DDPTA being peeled from adherends at different temperatures. e) Oscillatory rheology tests showing the temperature‐modulus curves of the DDPTA at different SA/TA ratios. f) Transmittance of a DDPTA patch after long‐term bonding at 32 °C. g) Hemolysis ratio and h) hemolysis photos of rat blood challenged with different wound patches (n = 3). i) Bars represent the concentration of immunoglobulin E (IgE, an allergic reaction marker) in the blood of the rats after the in vivo skin compatibility test of different wound patches (n = 3). j) In vivo skin compatibility test of different wound patches.

The on‐demand adhesion/removal property of the DDPTA is attributable to the semicrystalline‐to‐amorphous transition triggered by skin temperature. In the DDPTA, SA has a long alkyl side‐chain with a melting temperature between 47 °C and 50 °C.^[^ ^23^ ^]^ The addition of TA, with a shorter alkyl chain, reduces the phase‐transition temperature to below human skin temperature. Crystalline aggregates of TA and SA moieties act as hard segments, forming rigid phases at room temperature with no ability to adhere. As the semicrystalline SA melts at skin temperature, the rigid polymer becomes viscous and soft, capable of adhering to the wound (**Figure** **2**a–c). In contrast to the semicrystalline state of the DDPTA (at room temperature, 20 °C), the amorphous state (at human body temperature, 32 °C) is highly fluid and can easily be attached to the skin surface (Figure 2d, Figure S1, Supporting Information). To further understand this phase transition, we employed variable‐temperature X‐ray diffraction to explore the crystallization behavior of DDPTA at 20 °C and 32 °C (Figure S2, Supporting Information). At 20 °C, DDPTA showed a typical (100) diffraction peak at 21.4°, indicating its semicrystalline property. When the temperature reaches 32 °C, the intensity of the characteristic peak decreases, and the peak finally shifts to 19.1° with a wider width, which means that the semicrystalline phase dissociates into an amorphous phase. Moreover, because of the flexible alkyl chain, the DDPTA dissipates energy readily, ensuring that the tensile force is evenly distributed during removal or deformation, thereby reducing the risk of cracking.^[^ ^24^ ^]^

In the FTIR spectra of DDPTA, the disappearance of the absorption peak located at 1620 cm^‐1^ was associated with the C=C stretching vibration of SA, and TA indicates that acrylic monomers reacted with UD by free‐radical copolymerization (Figure S3, Supporting Information). To obtain the optimal transition temperature of the DDPTA, we prepared a series of samples with different SA:TA weight ratios and characterized their dynamic temperatures by rheometry. The semicrystalline‐to‐amorphous transition temperature of the DDPTA increases with an increase in SA doping ratio owing to the longer alkyl chain of SA (Figure 2e). At a SA:TA ratio of 0.8:1.2, the resulting DDPTA has a narrow transition temperature range between 25 °C to 29 °C, just below body surface temperature, making it an ideal candidate for on‐demand adhesion/removal on wounds. Patches with long‐lasting optical transparency are favorable in practical applications, making wound healing easier to observe at any time. We, therefore, measured the transmittance of DDPTA at different temperatures in the visible range (400–800 nm). The DDPTA was opaque at room temperature (Figure S4, Supporting Information) and became transparent at body temperature (Figure 2f), with average transmittances of 0.77% and 90.27%, respectively. This reversible change in transmittance occurs because DDPTA is opaque in the semicrystalline state (owing to Rayleigh scattering) and transparent in the amorphous state. The transmittance can remain at 88.84% even after one month, so it is basically stable during long‐term adhesion.

The biosafety of a wound patch is particularly important owing to its close contact with blood and skin during use. With this in mind, we comprehensively evaluated the biocompatibility of the DDPTA in vitro (hemocompatibility) and in vivo (skin compatibility), selecting gauze and Tegaderm (a commonly‐used commercial dressing) as controls. After co‐incubation with the above patches for 2 h at 37 °C, the supernatants from centrifuged blood samples were light yellow, similar to the control group (PBS), while the positive group (water) was bright red (Figure 2h). All of the hemolysis rates were calculated as less than 5%, proving satisfactory hemocompatibility (Figure 2i). For the in vivo skin compatibility test, 10 × 10 mm gauze, Tegaderm, and DDPTA were attached to rat skin for 24 h. Compared with the skin under the control (blank), gauze, and Tegaderm, the skin in contact with the DDPTA appeared normal, with no signs such as erythema or itching (Figure 2j and Figure S5, Supporting Information). Meanwhile, immunoglobulin E (IgE, an allergic reaction marker)^[^ ^25^ ^]^ in rat blood was monitored after the in vivo skin compatibility test, and the results confirmed there were no allergic reactions (Figure 2i). Moreover, hematoxylin‐eosin (HE) staining to reveal pathological changes in the skin in each group indicated that the DDPTA induced no tissue injury (Figure S6, Supporting Information). These results show that our DDPTA is a biocompatible tissue adhesive with a definite potential for clinical medical application.

**Figure 3:**
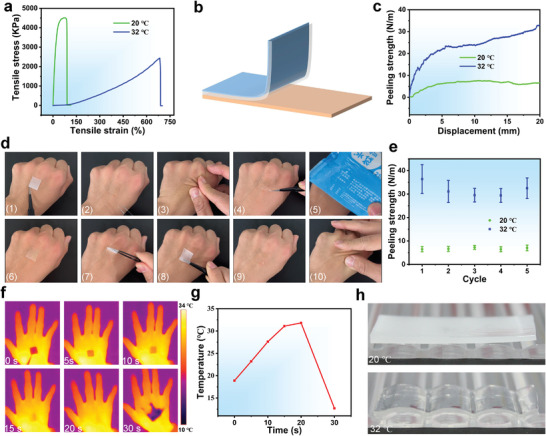
a) Tensile stress‐strain curves of the DDPTA at 20 °C and 32 °C. b) Schematic illustration of the standard 90°‐peeling‐off test. c) Peeling strengths of the DDPTA at 20 °C and 32 °C. d) Detailed process of on‐demand adhesion/removal and secondary adhesion of DDPTA on the hand skin. e) Cyclic peeling strengths of the DDPTA at 20 °C and 32 °C (n = 3). f) Real‐time infrared thermal images and g) temperature curve of the DDPTA on the back of the hand. h) Photographs of the DDPTA on a grooved surface at 20 °C and 32 °C.

A uniaxial tensile test showed a typical tensile stress‐strain curve of the DDPTA and was used to measure its mechanical strength (**Figure** **3**a). At room temperature (≈20 °C), the tensile stress of the DDPTA at the maximum tensile strain (81.7%) is 4504.3 KPa. At 32 °C the DDPTA is easy to flex and stretch, with ultimate tensile stress of 2434.6 KPa at a strain of 683.7%. The results confirm the stretchability of the DDPTA, making it suitable for wound sites with large deformation. Subsequently, its adhesion properties were examined on the skin using 90°‐peeling‐off tests (Figure 3b). After cooling with an ice pack, the adhesive strength of the DDPTA decreased dramatically from 33.1 N m^‐1^ to 7.5 N m^‐1^ (Figure 3c), ensuring no injury to the wound or skin when it was removed.

The on‐demand adhesion/removal and secondary adhesion of the DDPTA on the skin surface were monitored in detail (Video S1 and Figure 3d , Supporting Information). In a typical implementation, the DDPTA is relatively hard and opaque at room temperature and easy to handle (1); once in contact with the hand, it adheres to the skin within 10 s and becomes transparent owing to the semicrystalline‐to‐amorphous transition triggered by skin temperature (2); it can fit closely even when kneaded (3) and cannot easily be peeled off (4); upon challenging with an ice pack for 10 s (5), the DDPTA on the skin transitions to a semicrystalline state (6), then it becomes hard again and can easily be removed from the skin with no residue (7); after the skin returns to normal temperature, the removed DDPTA can re‐adhere and be re‐used (8–10). To explore the stability of on‐demand adhesion/removal, the peel strengths of the DDPTA at 20 °C and 32 °C were measured six times each. The peel strength at 32 °C varied slightly between cycles but remained high, but it was low at 20 °C (Figure 3e). An infrared thermal imager was also used to track the temperature‐triggered on‐demand adhesion/removal of the DDPTA (Figure 3f). Upon contact with the skin, the DDPTA reached the critical point of the semicrystalline‐to‐amorphous transition within 5 s, and then the temperature continued to rise, reaching skin temperature within 20 s. Upon treatment with an ice pack for 10 s, the temperature of the DDPTA rapidly decreased to 12.7 °C and its state transition was completed (Figure 3g). It was also ultra‐soft in the amorphous state, making it compatible with various parts of the body (Figure S7, Supporting Information). At room temperature (20 °C), it is rigid and always maintains its original planar shape with a large gap on its grooved surface. In sharp contrast, it could fit smoothly and tightly along the grooved surface at a higher temperature (32 °C), and there were no air bubbles between it and the surface (Figure 3h). Taken together, these results show that the DDPTA is an ideal wound patch in view of its unique on‐demand adhesion/removal triggered by skin temperature/ice pack, tunable transition temperature, and narrow temperature transition range (4 °C). At skin temperature, its peel strength is sufficient for intimate contact and adhesion with human skin in daily life, making it efficient as a wound patch. After cooling, the DDPTA patch could detach easily, preventing secondary injury to the wound during its replacement. More importantly, its state transition at ordinary and controllable temperatures ensures that it can be pasted repeatedly, so an incorrect pasting caused by operating errors in real applications can be corrected.

**Figure 4:**
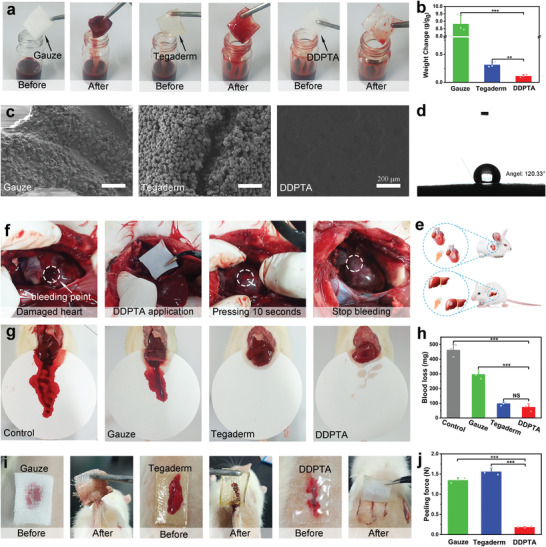
a) Photographs and b) weight changes of gauze, Tegaderm, and DDPTA before and after immersion in rat blood (n = 3). c) SEM images of gauze, Tegaderm, and DDPTA after immersion in rat blood. d) Contact angles of water on the surface of the DDPTA. e) Schematic illustrations of hemostasis in rabbit heart and rat liver injuries. f) DDPTA for closing and sealing heart‐penetrating injury in rabbits. g) Qualitative bleeding images and h) hemostatic efficacy (n = 3) of gauze, Tegaderm, and DDPTA. i) Pictures of wounds covered with gauze, Tegaderm, and DDPTA and peeling after 24 h. j) The average maximum peeling forces of gauze, Tegaderm, and DDPTA (n = 3). P‐values were determined by one‐way ANOVA. Statistical analysis was performed with the Student's t‐test. p < 0.001 (***), p < 0.01 (**), p > 0.05 (No significant, NS).

For hemorrhagic wounds, an effective treatment is to use gauze or a hemostatic dressing to press the wound mechanically, which inevitably results in additional blood loss and sticking of the hemostatic material to the wound owing to the absorption of excess blood.^[^ ^26^, ^27^ ^]^ The hydrophobicity of the DDPTA ensured by its long alkyl chains is conducive to avoiding the serious problems of additional blood loss and strong wound adhesion that plague hydrophilic materials in hemostatic applications. To evaluate the blood repellency of surfaces, we exposed three patches – gauze, Tegaderm, and DDPTA – to fresh rat blood for 1 min and compared their actions (**Figure** **4**a). Gauze immediately absorbs a large amount of blood, so it causes unnecessary blood loss and even endangers the lives of the wounded.^[^ ^28^ ^]^ Tegaderm is strongly hydrophilic so a thick layer of blood adheres to its surface, so it cannot avoid adhesion to the wound after hemostasis. The hydrophobic surface of the DDPTA resists blood contamination, effectively preventing blood infiltration and adhesion. In addition, the weight changes in gauze, Tegaderm, and DDPTA exposed with fresh rat blood were respectively recorded as 8.82, 0.31, and 0.11 g/g (Figure 4b). Furthermore, scanning electron microscopy (SEM) (Figure 4c) showed that a dense layer of blood cells adheres to the gauze surface, and more blood cells fill the interior owing to water absorption by the gauze. Multiple layers of blood cells were stacked on the Tegaderm surface and showed strong adhesion. However, no blood cells were attached to the DDPTA surface owing to its hydrophobicity. The liquid repellencies of gauze, Tegaderm, and the DDPTA were characterized by contact angle measurements (Figure 4d and Figure S8, Supporting Information). The water contact angles of gauze, Tegaderm were 17.76°, and 15.12° respectively. Contact angles of water and blood on the DDPAT surface were 120.33° and 104.5°, which further corroborated the above results regarding blood rejection.

Considering its stickiness and hydrophobic surface, the DDPTA is a promising tool for hemostasis. As a proof of concept, we validated its feasibility for closing and sealing cardiac penetrating injury in rabbits and liver injury in rats (Figure 4e). The surgical procedure is shown in Figure 4f. A 20‐gauge needle pierced the rabbit's heart, causing high‐pressure expulsion of blood. After the injury was covered with the DDPTA, cardiac bleeding stopped completely within 10 s, and even after 10 min under ambulatory blood pressure flow the DDPTA remained in place. To evaluate the in vivo hemostatic ability of the DDPTA, a rat liver hemorrhage model was tested (Figure 4g,f). Without hemostatic treatment, the injured liver left a large bloodstain and eventually 460.5 mg of blood was lost. The bleeding liver treated with gauze lost 295.2 mg of blood; the hemostatic efficiency was only ≈35.9%. After covering with Tegaderm, the hemostatic efficiency was significantly increased (79.1%), but some blood still adhered to its surface because it is strongly hydrophilic. Notably, the DDPTA stopped liver bleeding rapidly and completely and achieved 84.3% hemostatic efficacy. The excellent wound closure and hemostatic effects of the DDPTA were attributable to two factors: first, the dense DDPTA forms a physical barrier on the wound surface, preventing the penetration of blood; second, its hydrophobicity from the long alkyl chains effectively prevents blood infiltration and adhesion, leading to a partial reduction of blood loss. This hemostatic DDPTA patch is potentially valuable for rapid hemostasis and first aid to the wounded in emergencies.

Common patches such as gauze and Tegaderm absorb blood to form a super‐adhesive clot that requires forced peeling when they are replaced, which can reopen the wound and cause pain and secondary bleeding. In sharp contrast to existing hydrophilic patches, the DDPTA has the advantages of density, hydrophobicity, and on‐demand adhesive removal, and can be readily removed from the wound. A bleeding model on the rat's back verified these characteristics (Figure 4i and Figure S9, Supporting Information). The gauze absorbed a large amount of blood immediately upon contact with the wound, and most of its area was infiltrated with blood after 24 h. Because the gauze adhered strongly to it, the wound was pulled during peeling, resulting in severe tearing. After the wound was covered with hydrophilic Tegaderm, blood spread around it, and darkened blood clots were observed under the Tegaderm after 24 h. These clots disintegrated into particles during forced peeling and again caused severe tearing and secondary injury to the wound. In contrast, blood could be observed through the transparent DDPTA patch being repelled around the wound owing to the hydrophobicity, and no obvious blood clot was generated. With the aid of the ice pack, the DDPTA on the wound loses its stickiness and is easily removed without pulling the wound, preventing tearing and eliminating secondary bleeding.

The peel force required to remove the three patches was also measured on the rat's back (Figure 4j and Figure S10, Supporting Information). Owing to the irregular shape of the blood clot and the severe tearing of the wound during peeling, it is difficult to measure the width of contact between the material and the wound accurately. Therefore, the maximum force generated during peeling (averaged over three measurements) was used to compare the abilities of the different patches to reopen the wound quantitatively. The peeling forces of gauze and Tegaderm were similar, with average maxima of 1.35 and 1.56 N, respectively. The average maximum peeling force of the DDPTA was 0.180 N, 7.5 and 8.67 times lower than those for gauze and Tegaderm, respectively.

The in vitro and in vivo findings show the significant advantages of our DDPTA patch, which successfully overcomes the serious drawbacks of traditional hydrophilic patches. The DDPTA patch synergistically combines three core features to address the above limitations: (1) Its dense surface prevents blood infiltration and reduces unnecessary blood loss; (2) Its hydrophobicity prevents the formation of blood clots and precludes wound reopening when it is removed; (3) It's on‐demand adhesion/removal triggered by temperature can prevent the wound from being pulled by the adhesive. Given its unique wound detachment ability, the DDPTA has the potential to replace some clinical dressings, providing a promising solution for a wide range of secondary wound injuries.

**Figure 5:**
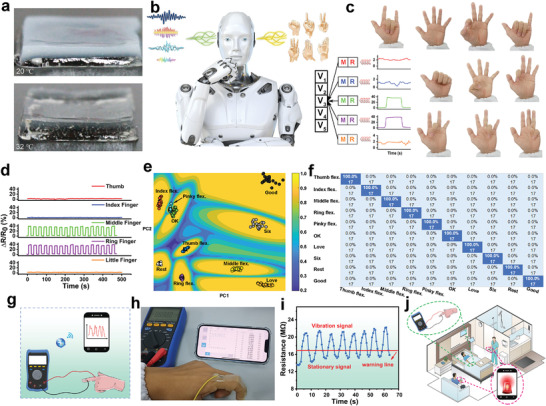
a) Photographs of DDPTA@ICE patch at 20 °C and 32 °C. b) Schematic illustration of machine learning algorithm used for gesture recognition. c) Photographs of ten different gestures recognized. d) Resistance signals of “Love” gestures. e) K‐means clustering and f) classification confusion matrix of the ten gestures. g) Schematic diagram and h) photograph of the external connections of the remote alarm system. i) Real‐time recording of finger‐bending movements received via Bluetooth and corresponding warning lines. j) Schematic diagram of the DDPTA@ICE system used for transmitting clinical information for language‐impaired wounded.

Extrapolating these advantages, the DDPTA can be also extended as an integrated platform by introducing other functional polymers^[^ ^29^ ^]^ to assist patients in gesture communication and remote alarm signaling. For this, we introduced a layer of stretchable and transparent ICE on the DDPTA surface to form a bilayer patch (i.e., DDPTA@ICE) with electrical conductivity, which supports subsequent sensing applications (**Figure** **5**a). As can be seen from Figure S11, (Supporting Information), the formed DDPTA@ICE patch displayed strong shape adaptability, as evidenced by the ability to withstand excessive twisting, bending, and stretching. Since its preparation does not use water as a solvent, ICE is very stable at body surface temperature without weight loss, which precludes the serious water loss of traditional hydrogels during sensing (Figures S12 and S13, Supporting Information). On the other hand, LiTFSI can easily be dissolved in various polymers and solvents and makes the ICE electrically conductive through ion transport by polymer chains, which becomes the key point of the DDPTA@ICE patch. The on‐demand adhesion/removal due to the crystalline phase‐transition of the DDPTA layer at different temperatures facilitates the use and replacement of a DDPTA@ICE patch on the skin surface. Since ICE is synthesized in‐situ on the DDPTA surface, the bonding is relatively tight, preventing the surface conductive layer from falling off during use. The DDPTA@ICE patch overcomes the disadvantages of traditional hydrogel patches that readily lose water, have low transparency, and are difficult to remove. On this basis, we further explored its application in gesture communication and remote clinical alarm signaling.

The resultant DDPTA@ICE is endowed with superior stretchability and flexibility. As depicted in Figure S14a, (Supporting Information), DDPTA@ICE can tolerate strain levels of more than 500%, ensuring stretchability for use on skin. Considering that most human strain range motions are less than 100%, such as bending wrists, knees, and finger joints, the loading‐unloading curves of DDPTA@ICE at 50%–90% strain were tested (Figure S14b, Supporting Information). The results show that DDPTA@ICE has a good correlation and reproducibility in this strain range. Importantly, the cyclic tensile‐strain curves of DDPTA@ICE (at 70% strain) remained almost unchanged after multiple loading/unloading cycles (Figure S14c, Supporting Information), indicating its reliability when applied to places with frequent movements such as joints. As the DDPTA@ICE patch sensor works under a resistance‐strain mechanism, the gauge factor value is critical to evaluate its sensitivity. The gauge factor of a strain sensor can be defined as (ΔR/R_0_)/(ΔL/L_0_). Figure S15, (Supporting Information) shows the different sensitivities of the DDPTA@ICE patch in three distinct strain stages. It was calculated to be 1.44, 1.52, and 2.03 in the strain range of 10–50%, 50–110%, and 110–200%, respectively.

As part of biomedical care, sign language recognition is essential for eliminating communication barriers between physicians and casualties who cannot communicate using spoken language. With the support of machine learning (Figure 5b), we propose a DDPTA@ICE‐based sign language recognition platform that can be attached/removed on demand, with the aim of lowering the communication barrier between physicians and casualties. Experimenters were asked to perform a total of 10 different gestures (Figure 5c), including five bendings and straightenings of individual fingers, and five common communication gestures. Each gesture was repeated 17 times to accumulate enough training samples. The resistive signals were segmented along a time coordinate, and two features [root mean square (RMS) and mean absolute value (MAV)] were extracted from the signal generated by each gesture movement to represent the response of the DDPTA@ICE patch array on the fingers. After the extracted eigenvalues were split into five subsets, fivefold cross‐validation was performed. The specific method is to use one of the subsets to establish a training model for testing while the other subsets are used as the training set, and the whole process is repeated five times. Concurrently, a k‐nearest neighbor classifier was used to give the percentage predicted classification as the accuracy of the corresponding gesture recognition without post‐processing. The corresponding resistance signals of these 10 gestures are shown in Figure 5d and Figures S16 and S17, (Supporting Information), indicating good signal reproducibility. Then machine learning recognizes all resistance signals and reversely reconstructs and outputs the corresponding predictions. After the five‐fold cross‐validation, the resulting confusion matrix shows that the prediction of gestures matches the actual gestures with 100% accuracy (Figure 5e,f), which demonstrates the feasibility of the DDPTA@ICE patch array assisted by machine learning for perceiving and classifying human gestures. Furthermore, this machine learning only requires a simple algorithm to analyze the data; complex data analysis processes and algorithms are not needed. This gesture recognition system could potentially be an auxiliary platform for sign language perception, and it will also be more important in non‐verbal clinical information transmission.

The popularization of smartphones gives them an irreplaceable role in clinical information transmission and data recording. Using mobile phones for real‐time signal transmission will be more convenient for medical diagnosis and wound care. Therefore, we supplemented the real‐time alarm system on the basis of sign language recognition to enhance the practical application of the DDPTA@ICE patch. A remote clinical alarm system consisting of a DDPTA@ICE patch, a smartphone, and a digital multimeter with Bluetooth was established for remote communication between the wounded and doctors (Figure 5g). Taking finger movement as an example, the digital multimeter collects the resistance change of the DDPTA@ICE patch during this process and transmits the data to the mobile phone APP via Bluetooth to monitor human movement in real time (Figure 5h). A threshold value was set in advance according to the resistance change of the patch. When the wounded's index finger is straightened normally, the system is stable, indicating that he or she does not need help. In the event of an emergency or need for help, he/she simply bends the fingers to make the resistance signal of the patch increase sharply and exceed the threshold, alerting doctors or family members through mobile phone vibrations (Figure 5i and Video S2, Supporting Information). This non‐verbal remote alarm system can serve as a bridge between the wounded and others, and will be important for transmitting clinical information (Figure 5j).

## 3 Conclusion

In this study, we have introduced a debonding‐on‐demand bilayer wound patch that could serve as a multifunctional platform for wound sealing and for clinical communication by the wounded. The semicrystalline‐to‐amorphous transition of the DDPTA between 25 °C and 29 °C makes it an ideal candidate for on‐demand adhesion/removal of wounds. The in vitro and in vivo test results show that it can effectively prevent blood infiltration and thus reduce unnecessary blood loss and the formation of blood clots, precluding wound tearing during replacement. The DDPTA was further covered with ICE to configure a bilayer DDPTA@ICE patch for building non‐verbal clinical communication systems for sign language recognition and remote alarm signaling by the wounded. The results show that the prediction of gestures matches the actual gestures with 100% accuracy, which demonstrates the feasibility of the DDPTA@ICE patch array assisted by machine learning in perceiving and classifying human gestures. The subsequently‐established remote alarm system consisting of a DDPTA@ICE patch, a smartphone, and a digital multimeter with Bluetooth enables the wounded to issue long‐distance non‐verbal emergency alarm signals. Considering these features and advantages of the DDPTA@ICE patch, it holds the potential to overcome the deficiencies of current functional materials and technologies and be widely used in various medical settings such as wound treatment and clinical communication.

## Conflict of Interest

The authors declare no conflict of interest.

## Supporting information

Supporting Information

Supporting Information

Supporting Information

## Data Availability

Research data are not shared.
