# Supplementary material for: Debonding‐On‐Demand Polymeric Wound Patches for Minimal Adhesion and Clinical Communication
Source: Adv Sci (Weinh). 2022 Aug 21;9(29):2202635. doi: 10.1002/advs.202202635 (PMC9561782; doi:10.1002/advs.202202635)
Supplement: Supplementary file 1 — Supporting Information [file ADVS-9-2202635-s002.pdf]

ADVANCED SCIENCE
Open Access

Supporting Information

for *Adv. Sci.*, DOI 10.1002/advs.202202635

Debonding-On-Demand Polymeric Wound Patches for Minimal Adhesion and Clinical Communication

Qiankun Zeng, Fangbing Wang, Ruixuan Hu, Xuyin Ding, Yifan Lu, Guoyue Shi, Hossam Haick and Min Zhang**

Supporting Information

Debonding-on-Demand Polymeric Wound Patches for Minimal Adhesion and Clinical Communication

Qiankun Zeng, Fangbing Wang, Ruixuan Hu, Xuyin Ding, Yifan Lu, Guoyue Shi, Hossam Haick, and Min Zhang**

Experimental Procedures

Materials and Reagents: Stearyl acrylate (SA), butyl acrylate (BA), lithium bis(trifluoromethane sulfonimide) (LiTFSI), polyethyleneglycol diacrylate (PEGDA) and 2,2-Dimethoxy-2-phenylacetophenone (DMPA) were purchased from Shanghai Aladdin Co., Ltd., (China). Tetradecyl acrylate (TA) was purchased from Tokyo Chemical Industry Co., Ltd., (Japan). 1-hydroxycyclohexyl phenyl ketone (photo-initiator 184) was purchased from Damas Beta (Shanghai, China). Rat IgE (Immunoglobulin E) ELISA Kit was purchased from Shanghai mlbio Biotechnology Co., Ltd., (China).

Preparation of DDPTA patch: DDPTA patch was prepared by copolymerizing a mixture of SA, TA, and UD via photo-polymerization. First, certain amount of SA (1.6 g), TA (2.4 g), UD (1g), and DMPA (30 mg) were mixed in a 10 mL flask. Afterward, the mixture was sonicated for 10 min and poured on a glass mold. The final DDPTA patch was obtained by UV polymerization (400 W, 365 nm, 10 min).

Preparation of DDPTA@ICE patch: DDPTA@ICE patch was prepared using the same method. Photo-initiator 184, PEGDA, BA, and LiTFSI were employed as photo-initiator, crosslinker, monomer, and electrolyte salt, respectively. First, photo-initiator 184, PEGDA, and LiTFSI were added into BA liquid, for complete stirring, and sonication. Next, the mixed solution was added to the mold containing the prepared DDPTA. Then, DDPTA@ICE patch with a bilayer structure was obtained by UV light irradiation (400 W, 365 nm, 10 min).

Rheological analysis: The rheological properties of sample were evaluated using a TA rheometer (DHR3) with a 25 mm parallel-plate configuration. Dynamic temperature sweep tests were carried out in the frequency of 1 Hz (10-60 ℃, 3 ℃/min). Transparency stability. The light transmittance of DDPTA at different temperatures and different adhesion times was tested by UV-vis spectrometer (UV-1800, Shimadzu, Japan) at wavelengths range of 400–800 nm.

Mechanical properties tests: Mechanical tests the samples (1.5 mm in thickness, 12 mm in width and 35 mm in length) were conducted on a tensile machine (HY-0580, Shanghai Hengyi Testing Instruments Co. Ltd., China). The stretching rate was set to 40 mm/min and a 50 N load cell was used. Peel strength.

To measure peel strength, adhered samples (1.5 mm in thickness, 12 mm in width and 35 mm in length) were prepared and tested by a standard 90° peeling testing using a tensile tester. The peel speed was set to 40 mm/min and a 50 N load cell was used.

***In vitro* hemocompatibility test:** Fresh whole blood from rats was centrifuged at 2000 rpm for 10 min, and washed with PBS for three times. Then, the precipitated red blood cells were diluted by PBS to a final concentration of 4% (v/v). Thereafter, the samples mixed with diluted blood were incubated at 37 °C for 2 h. PBS and deionized water were regarded as negative and positive controls, respectively. After that, the blood was centrifuged at 2000 rpm for 10 min, and the obtained supernatant was transferred to a new 96-well microplate. The absorbance of the supernatant was measured at 545 nm using a microplate reader (Infinite M200 Pro, Tecan, Austria) to determine the hemolysis percentage as reported previously.[1]

Hemostasis capacity evaluation: The hemostatic potential of samples in dynamic injuries was first studied by rabbit heart hemorrhage models. Male New Zealand white rabbit (2.5–3.0 kg) were anaesthetized with ear intravenous injection of 3% sodium pentobarbital (0.3 mL/100.0 g) and the abdomen was incised to expose heart. Then, a circular perforation wound was created on the rabbit heart by a 20 gauge needle to induce hemorrhaging. After quickly covering the bleeding site with the sample and pressing for ten seconds, the hemostasis effect was observed and recorded with a digital camera. To further evaluate the hemostatic ability of samples in vivo, rat liver hemorrhage models (male Sprague-Dawley rats, weight of 250–300 g) were employed. In brief, after anesthesia with 10% chloral hydrate (0.4 mL/100 g), the rat liver was exposed from an abdominal incision. Then, the liver was placed onto the pre-weighed filter paper and a 3 mm diameter injury was made using a biopsy punch (Dynarex). Hemostatic materials (n=3) with dimensions of 20 mm × 10 mm were immediately placed onto the surface of bleeding area to prevent bleeding. The total amount of blood loss until haemostasis was recorded by determining the total weight of hemostats and papers for each group.

Skin compatibility test: The skin compatibility was tested by attaching gauze, Tegaderm, TSU to the rat skin for 24 hours, and the size of all materials was 10*10 mm. After the rats were anesthetized and shaved, the prepared samples were immobilized on rat skin for 24 hours. 24 hours later, rats were anesthetized and checked for any differences between the areas under blank, gauze, Tegaderm and TSU. Immunoglobulin E (IgE) in blood of rats in each group were assessed by IgE ELISA assays (Mlbio, Shanghai, China). Finally, the rats were euthanized, and the skin pathological changes of each group were observed by hematoxylin-eosin (HE) staining.

Quantification of blood rejection: The same size (20 mm in length and 20 mm in width) of gauze, Tegaderm, DDPTA was immersed in fresh rat blood for 1 minute. At the end of the time, the material was weighed to calculate the blood entrapment, and this process was repeated three times. All samples were washed carefully by PBS to remove the physically adhered blood cells. Then, all samples were fixed

by 2.5 wt% of glutaraldehyde overnight, and gradually dehydrated by using a series of graded ethanol solution (50%, 60%, 70%, 80%, 90%, and 100%) with time interval of 10 min. After being dried at 37 °C for 12 h, they were cut and observed by SEM.

Peeling force test: Male Sprague-Dawley rats (250-300 g) were used for this study. After anesthesia with 10% chloral hydrate (0.4 mL/100 g), rats were placed over a thermal pad to prevent hypothermia, and hair on the back was removed. After disinfection with 75% ethanol, two skin incision (cut down to muscle, about 1 cm long) were made on the left and right sides of the back of the rat. The same size (20 mm in length and 20 mm in width) of Gauze (n = 3), Tegaderm (n = 3) and DDPTA (n = 3) were placed in the wound and fix. Peel force was measured (tensile machine) by peeling the samples along the wound after approximately 24 hours, allowing the clot to fully mature and coagulate. The maximum force generated during the peeling process (averaged over three measurements) was used to quantitatively compare the ability of different materials to damage the wound.

Statistical analysis: All quantitative results are shown as the mean ± standard deviation (SD) from at least three independent experiments. Statistical analysis was performed with the Student's t-test. Differences were considered statistically significant at $p < 0.05$ (*), $p < 0.01$ (**), $p < 0.001$ (***) and $p > 0.05$ (No significant, NS).

Note: All experiments with human research participants were approved by the University Committee of Human Research Protection (Approved number: HR 118-2022). All animal experiments were approved by the Animal Ethics Committee of East China Normal University (Approved number: R20210209). Study Participation: Prior to participation in the experiments, informed consent was obtained from the volunteer in all experiments.

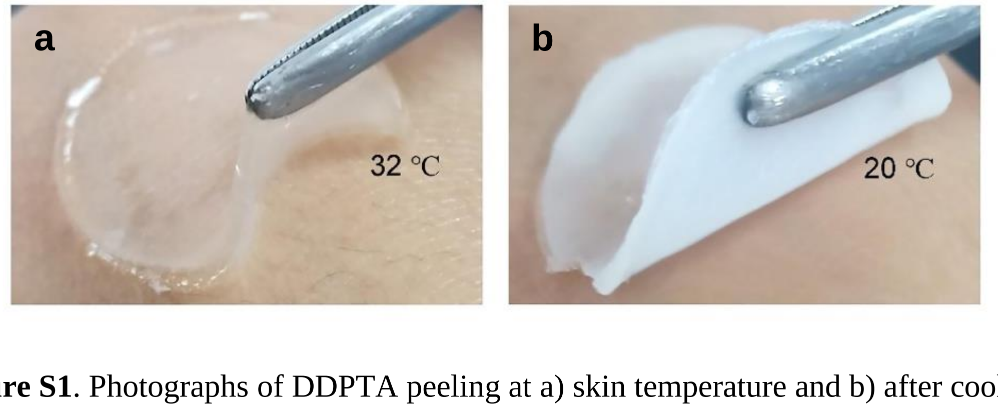

Figure S1. Photographs of DDPTA peeling at a) skin temperature and b) after cooling.

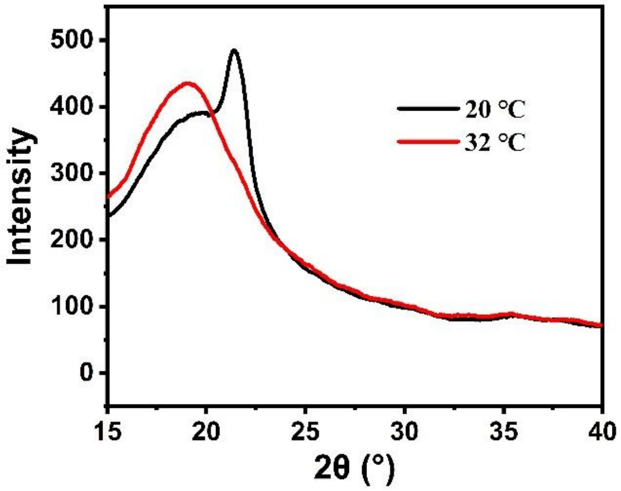

Figure S2. XRD patterns of DDPTA in 20 °C and 32 °C.

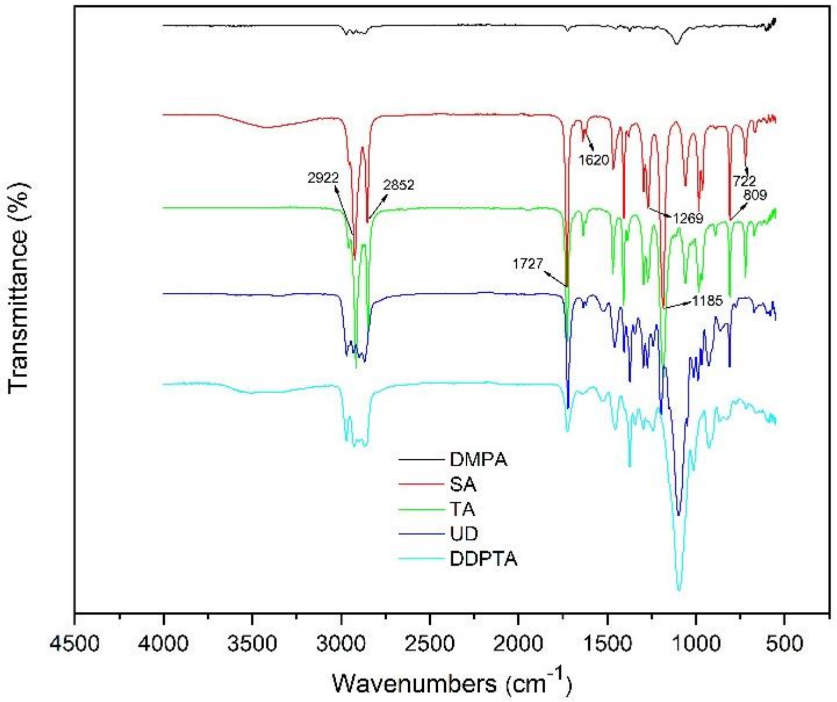

Figure S3. FTIR spectra of the monomers and DDPTA. In the spectra of the polymers, peaks with maxima at 2922 and 2852 cm^{-1} were associated with symmetrical and asymmetrical C–H stretching vibrations of typical CH_3– and –CH_2– absorption peaks. The characteristic peak at 1727 cm^{-1} was due to carbonyl group stretching (C=O) of ester group. The characteristic peak at 1269 and 1185 cm^{-1} were corresponded to the –C–O–C– stretching vibration peaks. Peaks at 809, and 722 cm^{-1} were attributed to the C–H bond and long-chain alkyl (–$(CH_2)_n$– and n greater than 4).

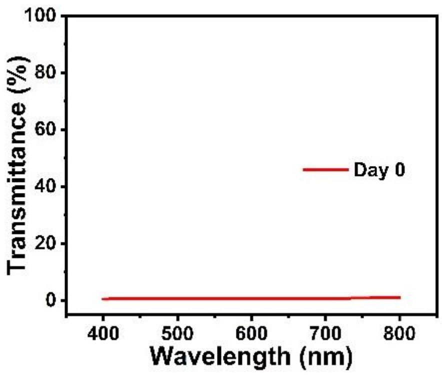

Figure S4. Transmittance of DDPTA patch under 20 ℃.

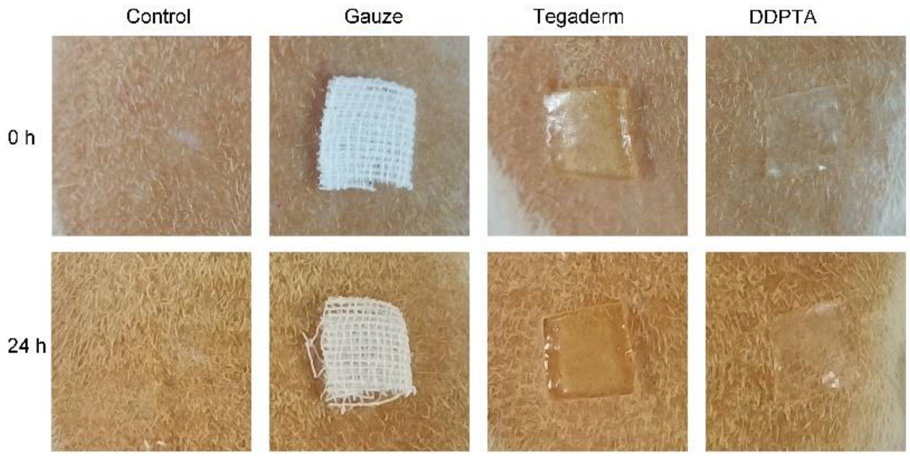

Figure S5. *In vivo* skin compatibility test.

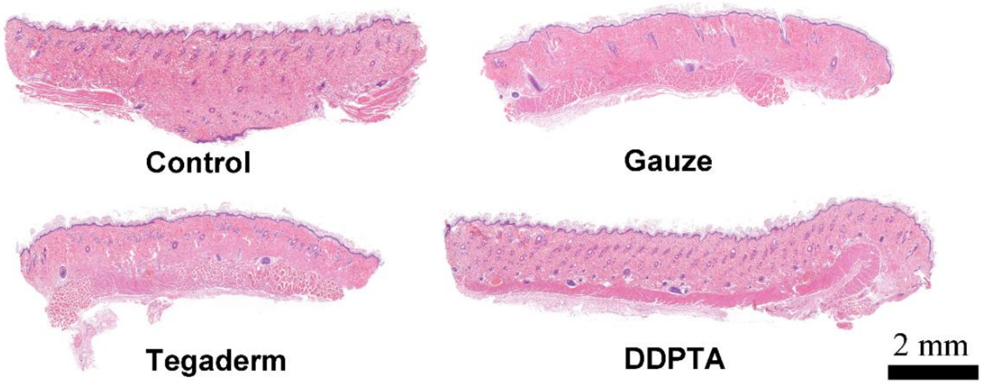

Figure S6. Hematoxylin-eosin (HE) staining of skin tissue in each group.

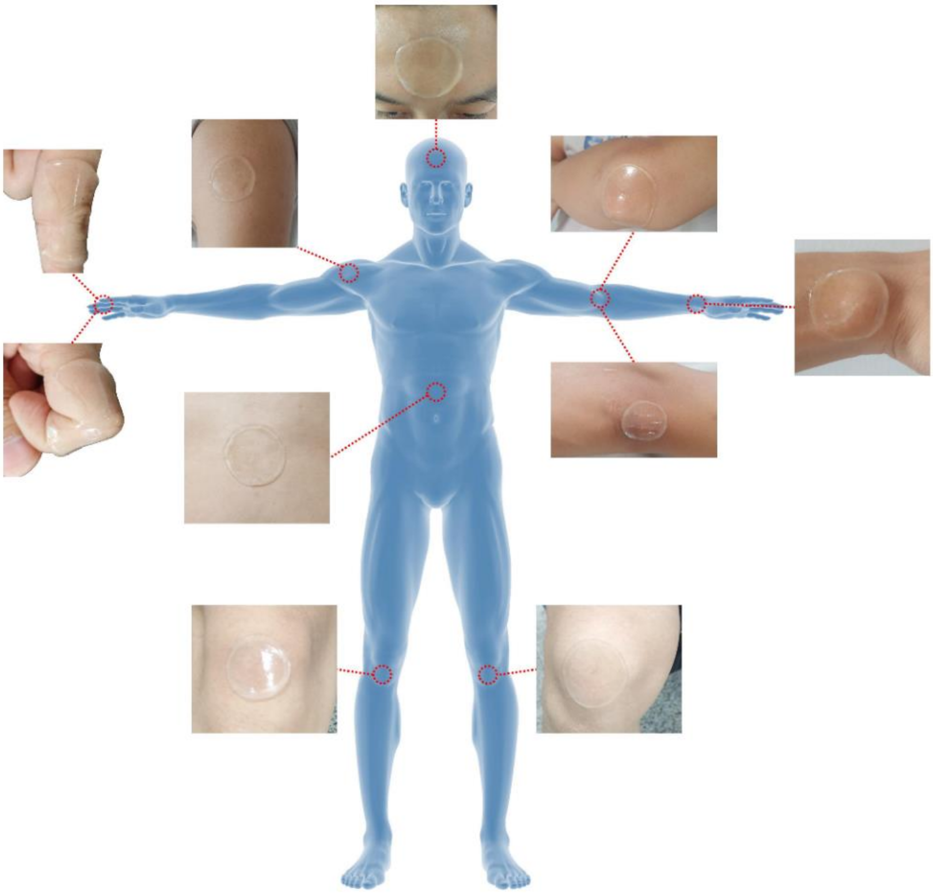

Figure S7. Photos of DDPTA sticking to various parts of the body.

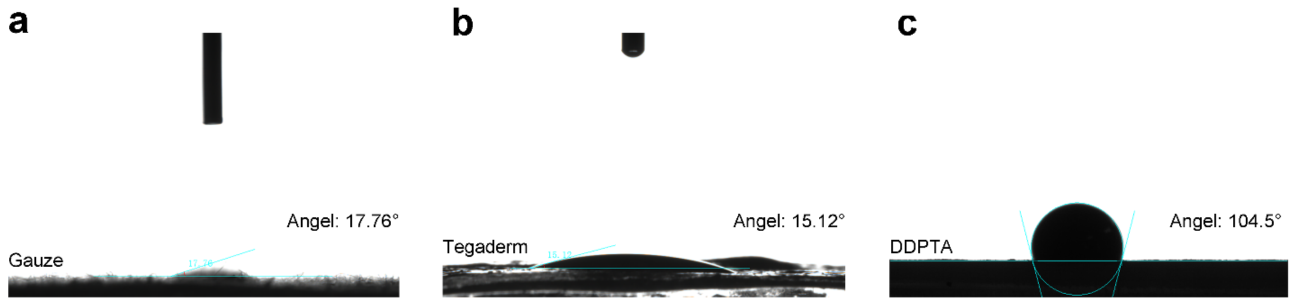

Figure S8. Contact angles of water on the surface of gauze (a) and Tegaderm (b), and contact angles of blood on the DDPAT surface (c).

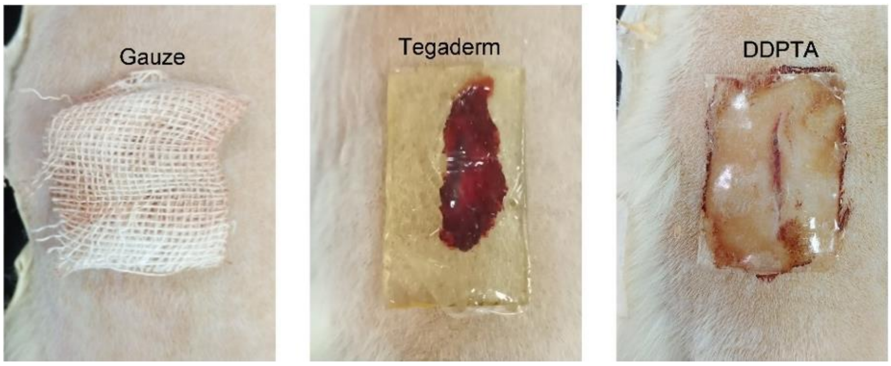

Figure S9. Photographs of the wounds covered with gauze, Tegaderm, and DDPTA for 24 hours.

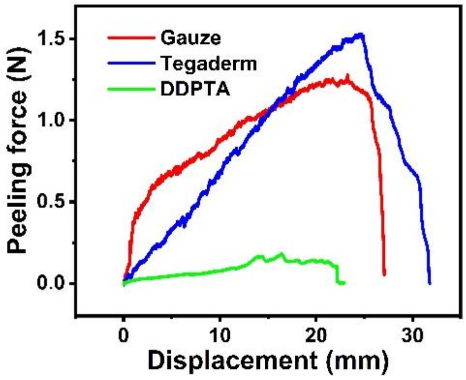

Figure S10. The peel force required for removal of the three patches from wound.

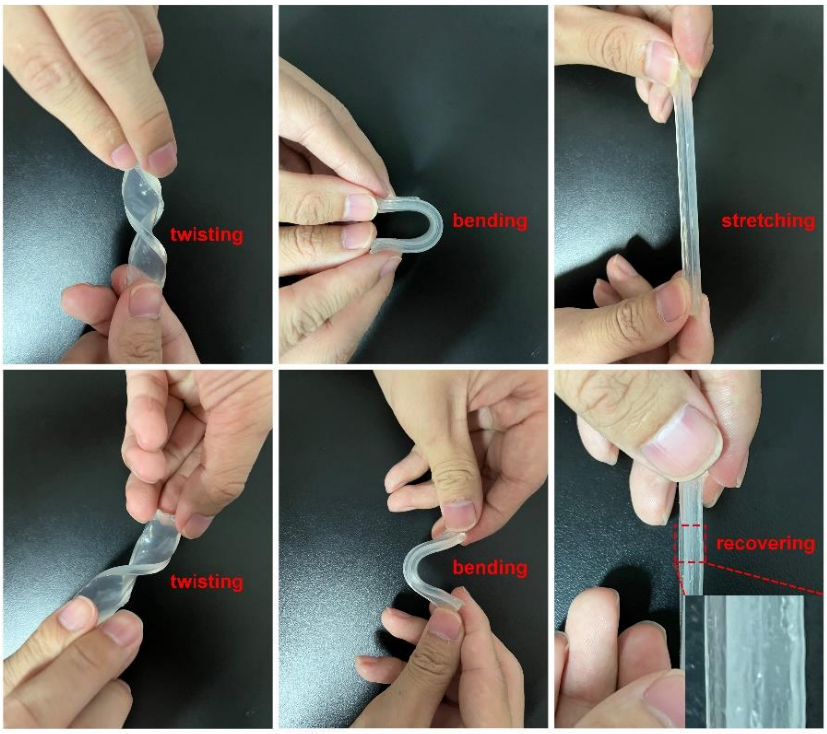

Figure S11. Photographs of the DDPTA@ICE patch recovering after being subjected to excessive twisting, bending, and stretching.

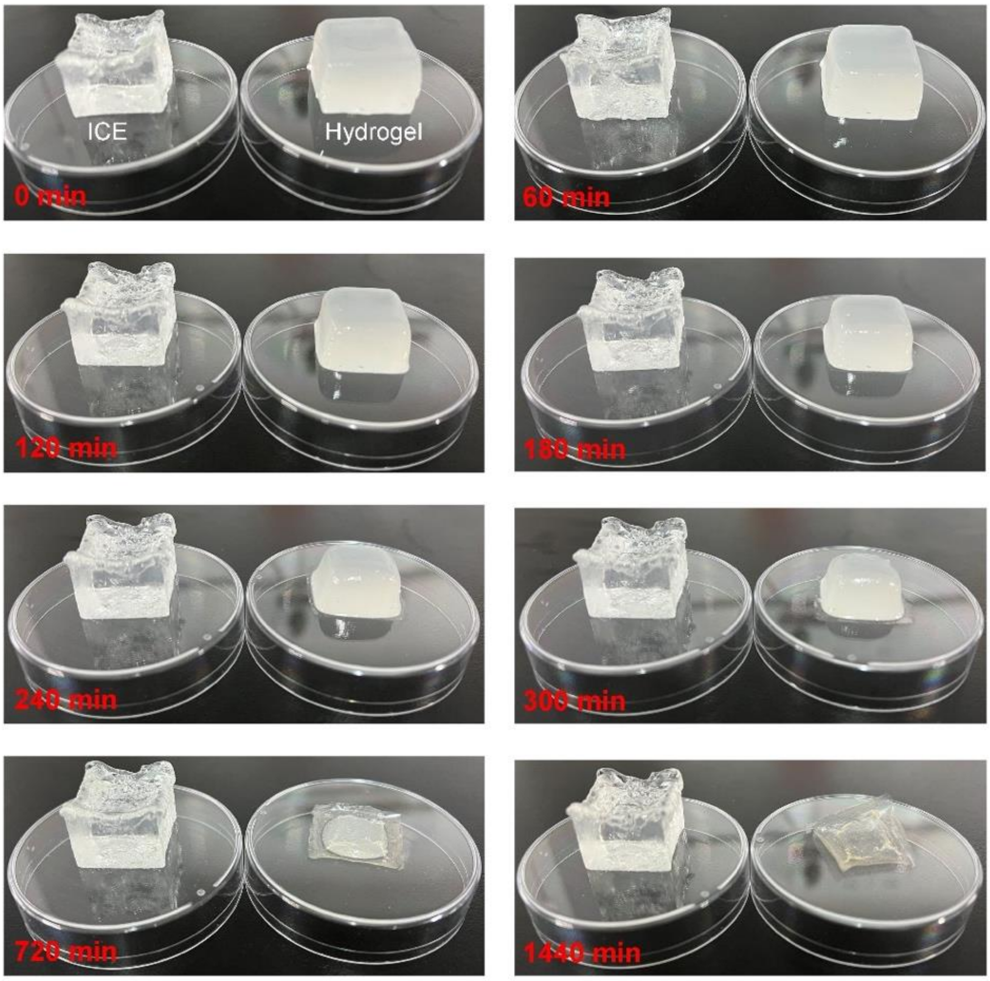

Figure S12. Photographs of ICE and hydrogel at 37°C for 24h.

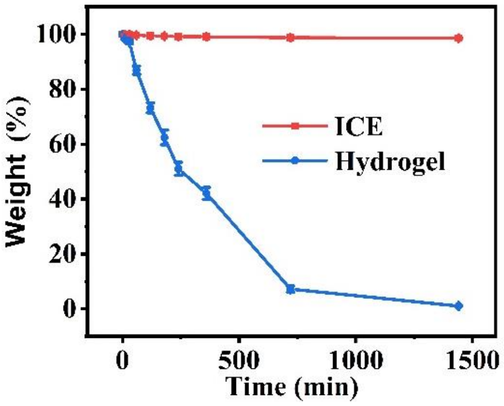

Figure S13. Weight changes of ICE and hydrogel at 37°C for 24h.

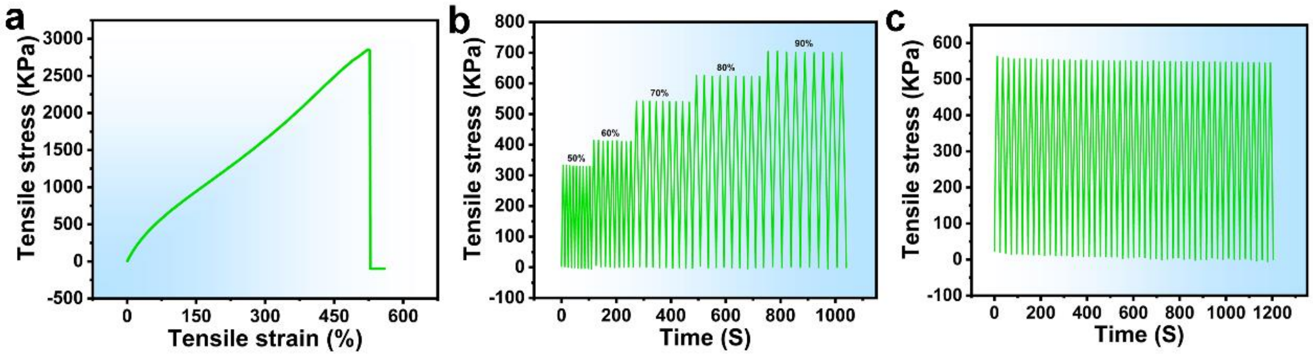

Figure S14. Mechanical property of DDPTA@ICE: fracture strength (a), loading-unloading curves under different strains (b) and cyclic tensile-strain curves at 70% strain (c).

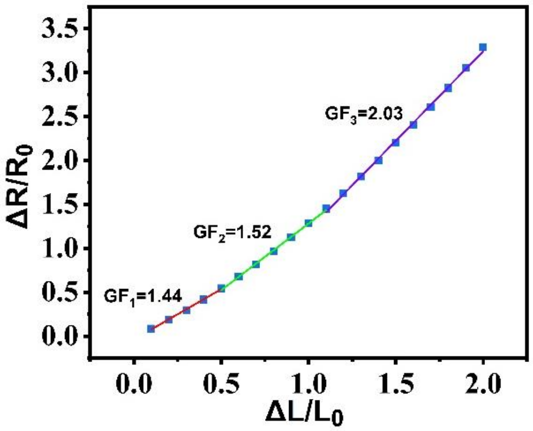

Figure S15. Gauge factors (GF) of DDPTA@ICE patch at various strains.

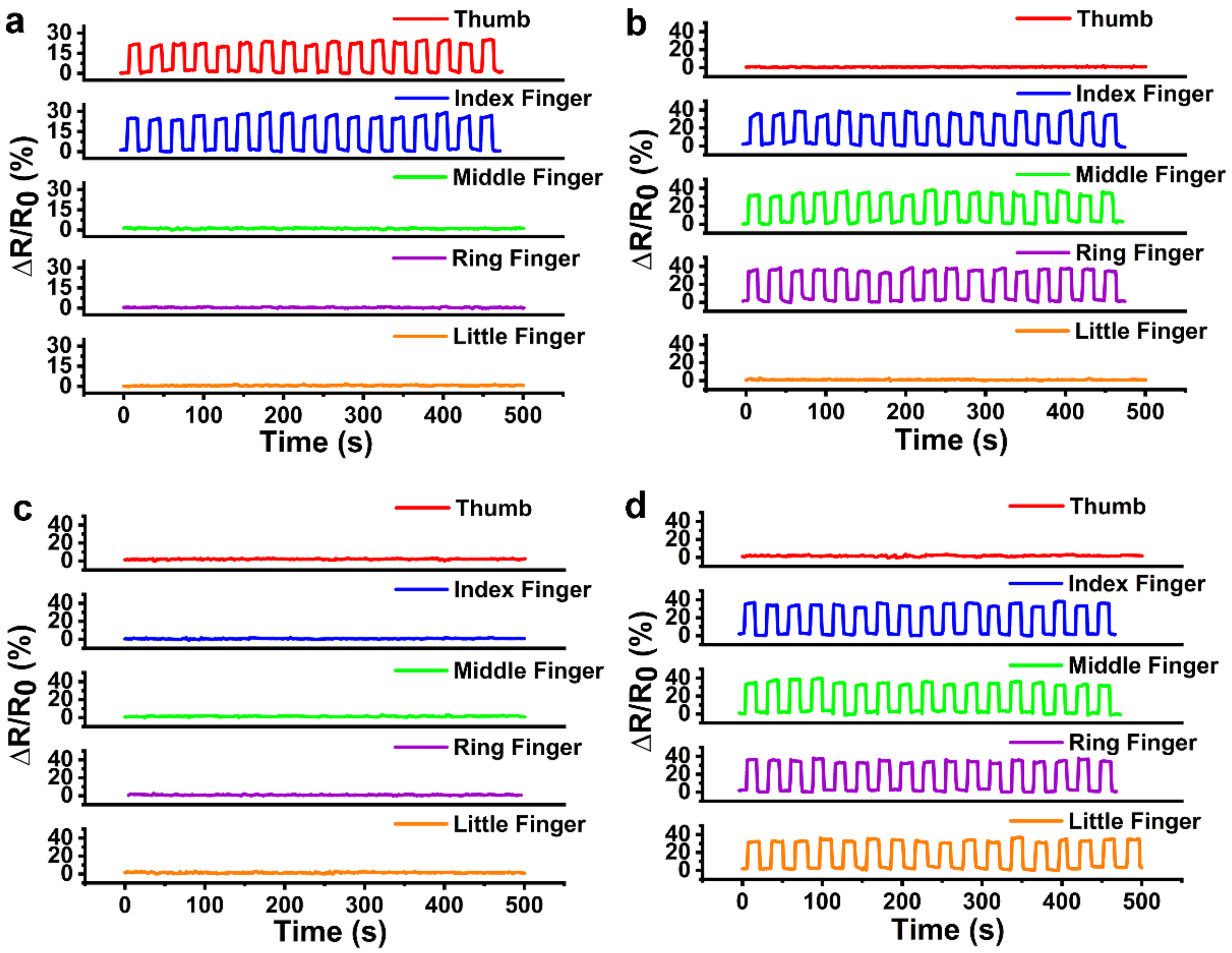

Figure S16. The resistance signals of a) OK gestures, b) Six, c) Rest, and d) Good.

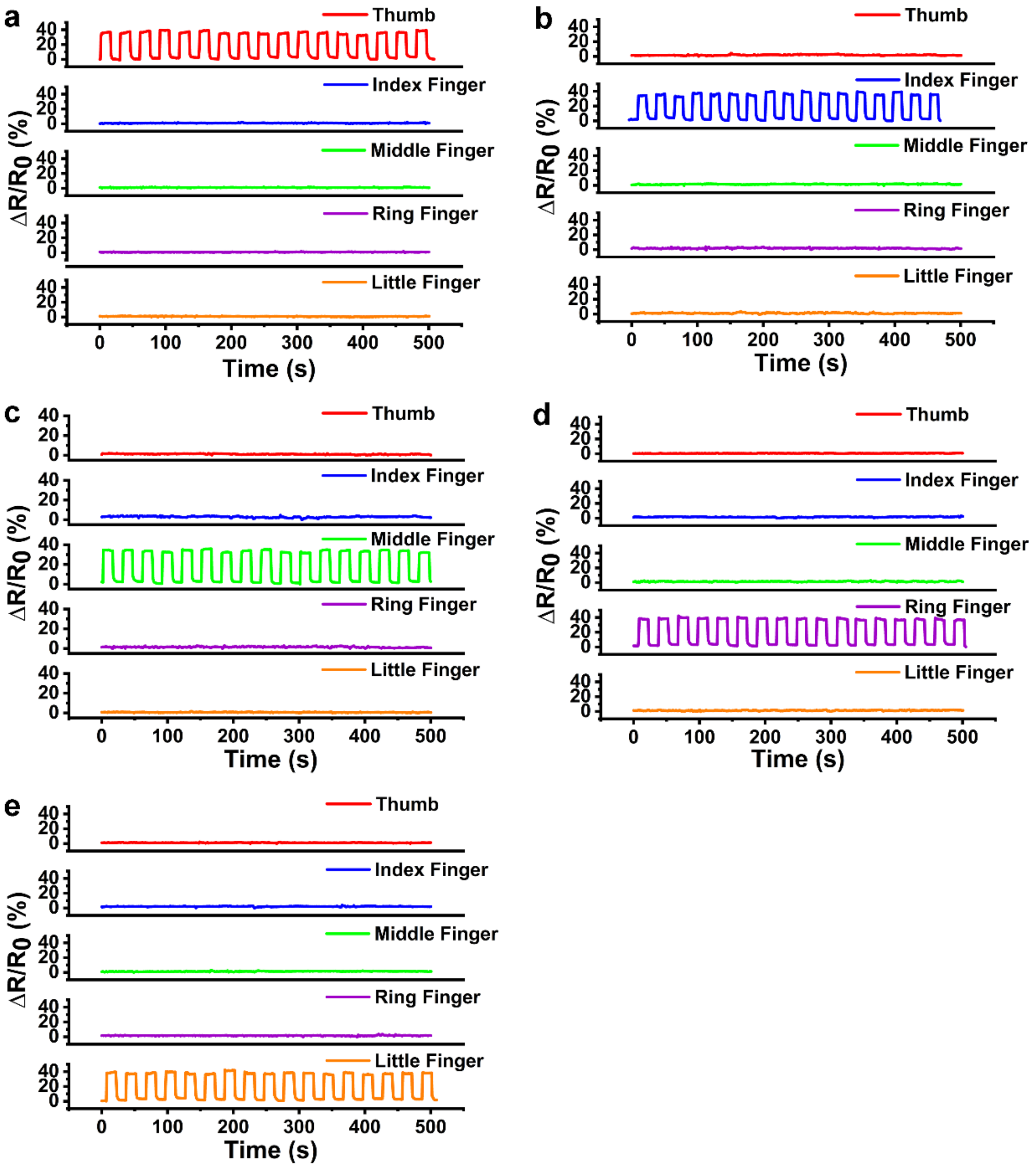

Figure S17. The resistance signals of a) Thumb inflect, b) Index inflect, c) Middle inflect, d) Ring inflect, e) Little inflect.
